# Predicting the microbial cause of community-acquired pneumonia: can physicians or a data-driven method differentiate viral from bacterial pneumonia at patient presentation?

**DOI:** 10.1186/s12890-020-1089-y

**Published:** 2020-03-06

**Authors:** Claire Lhommet, Denis Garot, Leslie Grammatico-Guillon, Cassandra Jourdannaud, Pierre Asfar, Christophe Faisy, Grégoire Muller, Kimberly A. Barker, Emmanuelle Mercier, Sylvie Robert, Philippe Lanotte, Alain Goudeau, Helene Blasco, Antoine Guillon

**Affiliations:** 1 CHRU Tours, Service de Médecine Intensive Réanimation, 2 Bd Tonnellé, F-37044 Tours Cedex 9, France; 2 CHRU Tours, Service d’Information Médicale, d’Epidémiologie et d’Economie de la Santé, Tours, France; 3 CHRU Tours, Laboratoire de Biochimie et Biologie Moléculaire, Tours, France; 4 CHRU Angers, Service médecine intensive et réanimation, Angers, France; 5 UPRES EA220, Laboratoire de recherche en pharmacologie respiratoire, Université Versailles Saint-Quentin, Suresnes, France; 6 CHR Orléans, Service de Médecine Intensive Réanimation, Orléans, France; 7 Pulmonary Center, Boston University School of Medicine, Boston, MA USA; 8 CHRU Tours, Service de bactériologie, virologie et hygiène hospitalière, Tours, France; 9 INSERM U 930, Université de Tours, Tours, France; 10 INSERM, centre d’étude des pathologies respiratoires (CEPR), U1100, Université de Tours, Tours, France

**Keywords:** Community-acquired pneumonia, Diagnosis, Artificial intelligence

## Abstract

**Background:**

**C**ommunity-acquired pneumonia (CAP) requires urgent and specific antimicrobial therapy. However, the causal pathogen is typically unknown at the point when anti-infective therapeutics must be initiated. Physicians synthesize information from diverse data streams to make appropriate decisions. Artificial intelligence (AI) excels at finding complex relationships in large volumes of data. We aimed to evaluate the abilities of experienced physicians and AI to answer this question at patient admission: *is it a viral or a bacterial pneumonia?*

**Methods:**

We included patients hospitalized for CAP and recorded all data available in the first 3-h period of care (clinical, biological and radiological information). For this proof-of-concept investigation, we decided to study only CAP caused by a singular and identified pathogen. We built a machine learning model prediction using all collected data. Finally, an independent validation set of samples was used to test the pathogen prediction performance of: (*i*) a panel of three experts and (*ii*) the AI algorithm. Both were blinded regarding the final microbial diagnosis. Positive likelihood ratio (LR) values > 10 and negative LR values < 0.1 were considered clinically relevant.

**Results:**

We included 153 patients with CAP (70.6% men; 62 [51–73] years old; mean SAPSII, 37 [27–47]), 37% had viral pneumonia, 24% had bacterial pneumonia, 20% had a co-infection and 19% had no identified respiratory pathogen. We performed the analysis on 93 patients as co-pathogen and no-pathogen cases were excluded. The discriminant abilities of the AI approach were low to moderate (LR+ = 2.12 for viral and 6.29 for bacterial pneumonia), and the discriminant abilities of the experts were very low to low (LR+ = 3.81 for viral and 1.89 for bacterial pneumonia).

**Conclusion:**

Neither experts nor an AI algorithm can predict the microbial etiology of CAP within the first hours of hospitalization when there is an urgent need to define the anti-infective therapeutic strategy.

## Background

The World Health Organization (WHO) estimates that due to antimicrobial resistance, bacterial infections will outcompete any cause of death by 2050 [1], meaning that there is an urgent need for new strategies to improve antibiotic treatments. The *Agency for Healthcare Research and Quality (AHRQ) Safety Program for Improving Antibiotic Use* recently proposed a structured approach to improve antibiotic decision making by clinicians, which emphasizes the 4 critical time points in antibiotic prescribing [2, 3]. The first time point of this organized approach requires the physician to ask: “Does this patient have an infection that requires antibiotics?”. This question aims to remind the clinician to synthesize all relevant patient information to determine the likelihood of an infection that requires antibiotic therapy. The questionable ability of physicians to answer this first question properly in the context of pneumonia was the impetus for this study.

Community-acquired pneumonia (CAP) is a major global healthcare burden associated with significant morbidity, mortality and costs [4–9]. Identifying the etiology of CAP is an utmost priority for its management and treatment decisions [10]. Although the range of pathogens that may be involved in these cases is broad, physicians must at least determine whether a bacterial or a viral pathogen (or both) is causing the pneumonia to determine if antibiotic treatment is appropriate. Whether the etiology of CAP is viral or bacterial should be determined based on the patient interview, clinical symptoms and signs, biological findings and radiological data from the very first hours of the patient’s presentation (a time when microbiological findings are typically not yet available). Physicians must use the knowledge obtained from their routine practice and medical education to make sense of these diverse data input streams, triage the resulting complex dataset, and make appropriate decisions. A growing body of research has recently suggested that difficulties in accessing, organizing, and using a substantial amount of data could be significantly ameliorated by use of emerging artificial intelligence (AI)-derived methods, which are nowadays applied in diverse fields including biology, computer science and sociology [11]. AI excels at finding complex relationships in large volumes of data and can rapidly analyze many variables to predict outcomes of interest. In the context of CAP in intensive care units (ICUs), where information are particularly diverse, we wondered if an AI data-driven approach to reducing the medical complexity of a patient could allow us to make a better hypothesis regarding the microbial etiology at the patient’s presentation.

The aim of our study was to evaluate and compare the abilities of experienced physicians and a data-driven approach to answer this simple question within the first hours of a patient’s admission to the ICU for CAP: *is it a viral or a bacterial pneumonia?*

## Methods

This study was conducted in two steps. First, we performed prospective data collection (step 1); second, we retrospectively assessed the microbial etiology prediction performances of experienced physicians (more than 10 years’ experience) and a computational data-driven approach for this dataset (step 2).

### Step 1: patient data collection

Prospective data collection was conducted in a single center over an 18-month period. The study complied with French law for observational studies, was approved by the ethics committee of the French Intensive Care Society (CE SRLF 13–28), was approved by the *Commission Nationale de l’Informatique et des Libertés* (CNIL) for the treatment of personal health data. We gave written and oral information to patients or next-of-kin. Patients or next-of-kin gave verbal informed consent, as approved by the ethic committee. Eligible patients were adults hospitalized in ICU for CAP. Pneumonia was defined as the presence of an infiltrate on a chest radiograph and one or more of the following symptoms: fever (temperature ≥ 38.0 °C) or hypothermia (temperature < 35.0 °C), cough with or without sputum production, or dyspnea or altered breath sounds on auscultation. Community-acquired infection was defined as infection occurring within 48 h of admission. Cases of pneumonia due to inhalation or infection with pneumocystis, pregnant women and patients under guardianship were not included. Cases with PaO2 ≥ 60 mmHg in ambient air or with the need for oxygen therapy ≤4 L/min or without mechanical ventilation (invasive or non-invasive) were not included.

Baseline patient information was collected at case presentation through in-person semi-structured interviews with patients or surrogates (see Supplementary Table 1). Observations from the physical examination at presentation, including vital signs and auscultation of the lungs, were recorded. Findings of biological tests done at presentation (within the first three-hour period) were also recorded (hematology and chemistry tests), as were findings from chest radiography. Two physicians interpreted chest x-rays; a third physician reviewed the images in cases of disagreements in interpretation.

Microbiological investigations included blood cultures, pneumococcal and legionella urinary antigen tests, bacterial cultures and multiplex PCR RespiFinder SMART 22® (PathoFinder B.V., Oxfordlaan, Netherlands) analyses on respiratory fluids (sputum and/or nasal wash and/or endotracheal aspirate and/or bronchoalveolar lavage [BAL]).

### Step 2: clinician and data-driven predictions of microbial etiology

**Fig. 1:**
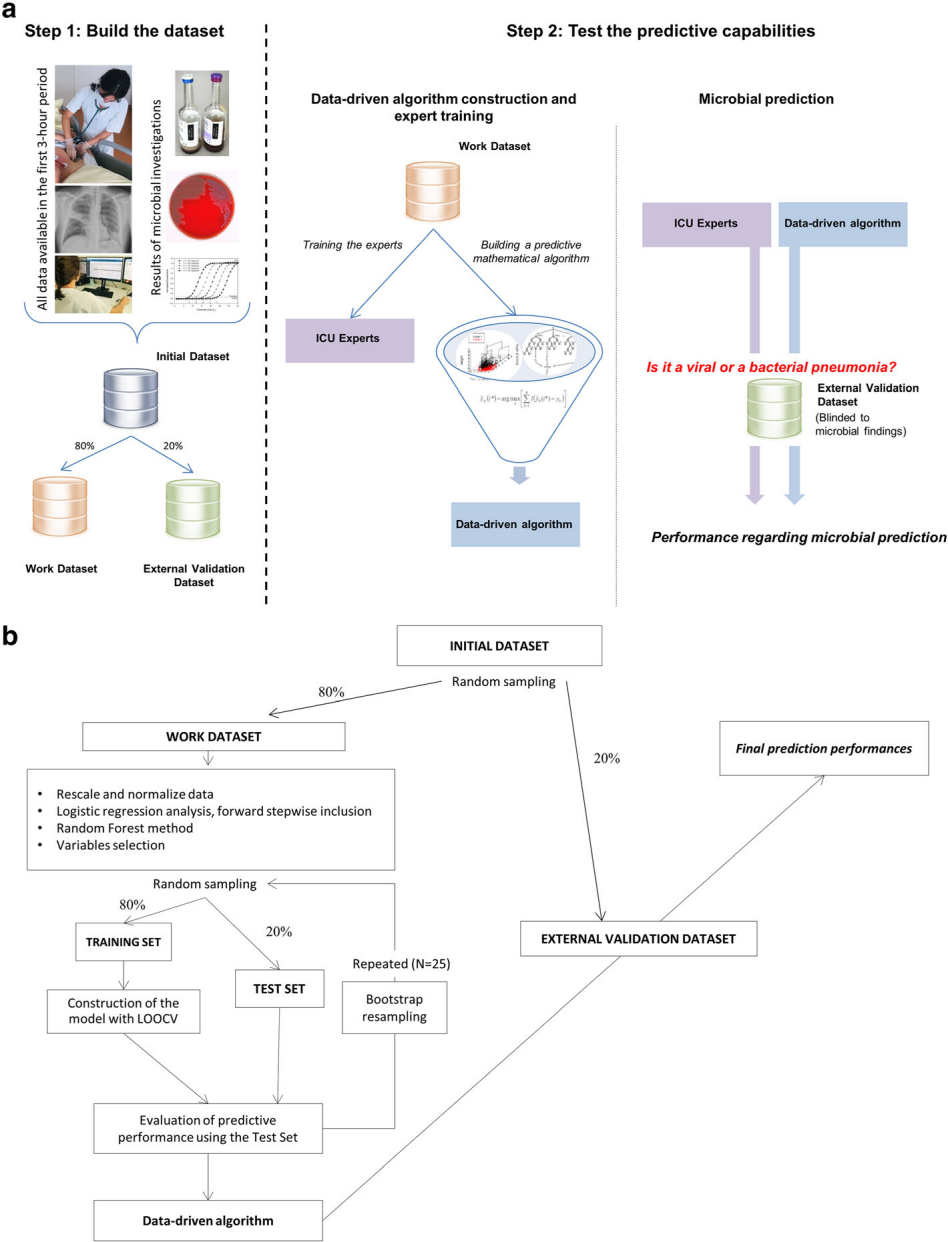
Schematic representation of the study methodology. **a** We built an *initial dataset* from all sources of information available in the first 3 h of the patient’s presentation in the ICU for CAP. We matched these presenting cases with their final identified causal respiratory pathogen. The *initial dataset* was randomly split into a *work dataset*, used for the machine learning and training the ICU experts on how the data were presented, and an *external validation dataset* used to assess the prediction performances of the artificial intelligence (AI) algorithm and the panel of experts. **b** Data flow to engineer the data-driven algorithm

Clinicians and a mathematical algorithm were tasked with predicting the microbial etiology of pneumonia cases based on all clinical (43 items), and biological or radiological (17 items) information available in the first 3-h period after admission except for any microbiological findings (Supplementary Table 1). For this proof-of-concept investigation, we decided to study only CAP caused by a singular and identified pathogen; cases of CAP with mixed etiology or without microbiological documentation were excluded. From the initial dataset of patients, we randomly generated two groups (prior to any analysis): (i) a work dataset (80% of the initial dataset) dedicated to construction of the mathematical model and training the experts; (ii) an external validation dataset (20% of the initial dataset) dedicated to testing the prediction performances. The methodology used is summarized in Fig. 1a.

#### Clinician predictions

An external three member expert panel reviewed the work dataset to familiarize themselves with the dataset containing the patient characteristics. Then, the experts were asked to predict the microbial etiologies in the external validation dataset (Fig. 1a). The clinicians had to answer the question: *is it a viral or a bacterial pneumonia?* They were also asked to give a confidence index regarding the accuracy of their answer: 1 (very low), 2 (low), 3 (moderate), 4 (high). Agreement of at least two of the three experts was required for the final predicted etiology.

#### Data-driven approach predictions

The data were analyzed using an AI method (Fig. 1b) involving a logistic regression analysis using forward stepwise inclusion. This method was employed to optimize the ability of the algorithm to distinguish viral and bacterial pneumonia based on the combination of parameters available in the work dataset. All available data were thus included in the model, regardless of the data type. Qualitative data were processed as binary information (i.e. influenza immunization: present “1”, absent “0”). Raw data were provided for quantitative values (no cut-offs defined). We built the predictive mathematical model from the work dataset using the Random Forest method and Leave-One-Out Cross-Validation. We started by determining the most relevant item to use through a variable selection procedure using the Random Forest method and the Mean Decrease in Gini criterion (value 0.75). Then, the population in the work dataset was randomly separated into two independent datasets: 80% of cases were assigned to the training set and 20% were assigned to the test set. N models with bootstrap resampling (with *N* = 25) were performed on the training set and validated on the test set. The model providing the best prediction criteria was selected, and the final model was built from the entire work dataset. Finally, an independent validation set of samples was used to test the pathogen prediction performance of the AI algorithm. To decipher the relative importance of clinical versus biological/radiological variables in the predictions, we generated three algorithms built from different parameters of the work dataset: (*i*) clinical variables only, (*ii*) biological and radiological variables only, and (*iii*) all variables. For each parameter tested, the area under the ROC curve (AUC) was calculated, and the best cutoff value that yielded the highest accuracy was determined along with the sensitivity and specificity.

### Statistical analysis

We compared the concordance between the predictions and the final microbial etiologies for the experts and for the algorithm and calculated sensitivity, specificity, positive predictive value (PPV), negative predictive value (NPV) and likelihood ratios (LRs) for the predictions [12]. Given the importance of this diagnostic prediction in the patient’s therapeutic management, we determined that the discriminant properties should be “high” (LR + > 10 and/or LR- < 0.1) for the prediction to be considered useful for clinical practice [13, 14]. Table 1 summarizes the LR cutoff values defining the discriminant properties of the predictions [13]. Quantitative data are reported as the median value and interquartile range (IQR). Statistical analyses were done with JMP software (SAS, version 7.2).

**Table 1:** Interpretation of likelihood ratios

LR+	LR-	Discriminant properties
> 10	< 0.1	High
5–10	0.1–0.2	Moderate
2–5	0.2–0.5	Low
1–2	0.5–1	Very low

LR+, positive likelihood ratio; LR-, negative likelihood ratio

## Results

**Fig. 2:**
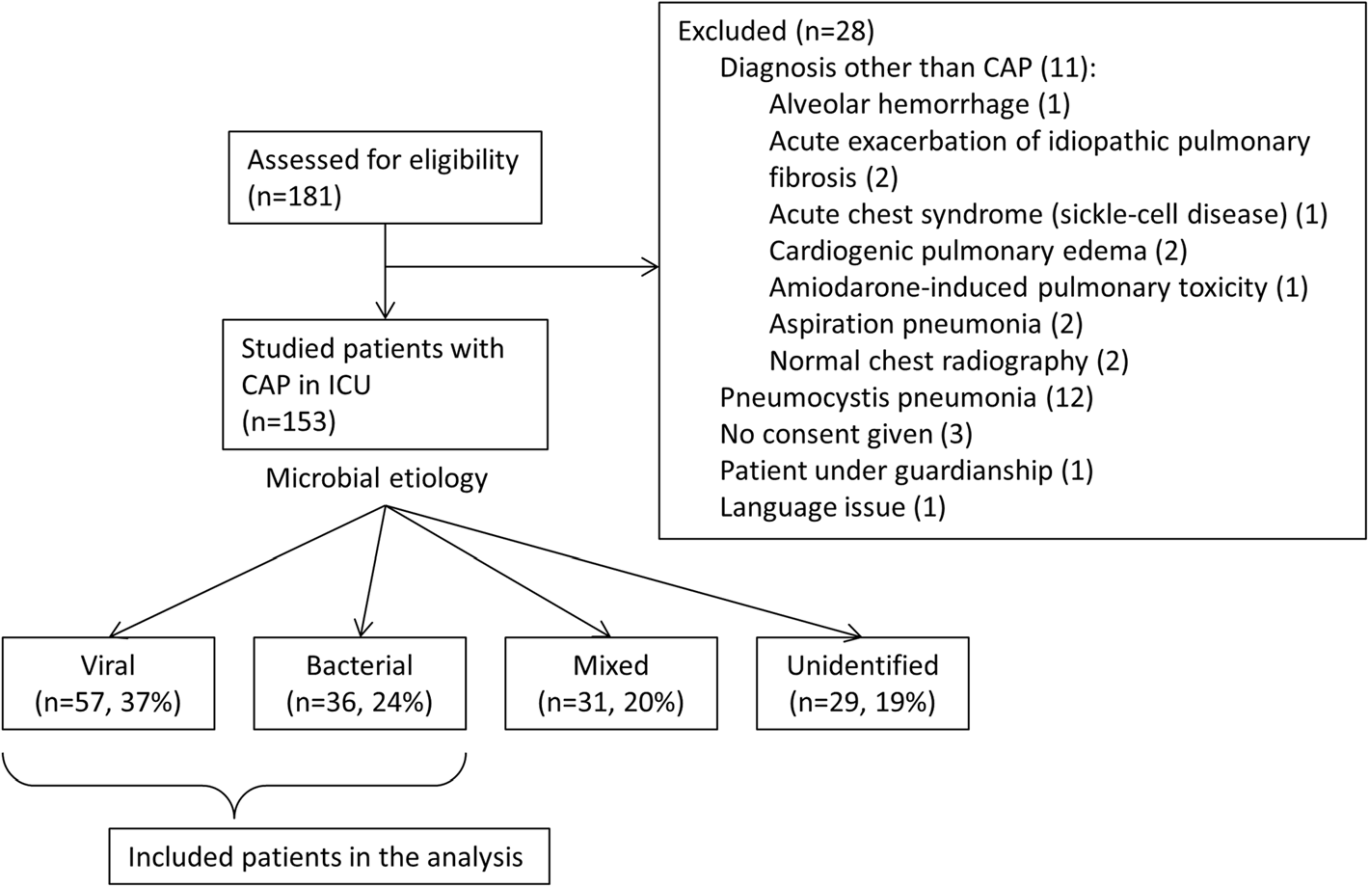
Flow chart for patient selection

A total of 188 patients diagnosed with CAP were eligible for inclusion over an 18-month period; 153 patients were included; 37% had viral pneumonia, 24% had bacterial pneumonia, 20% had a co-infection and 19% had no identified respiratory pathogen. Finally, we performed the analysis on 93 patients as co-pathogen and no-pathogen cases were excluded. The patient selection flow chart is presented in Fig. 2. The characteristics of the patients according to microbial diagnosis are detailed in Table 2.

**Table 2:** Baseline patient characteristics

	Total (***n*** = 153)	Bacteria (***n*** = 36)	Virus (***n*** = 57)	Co-infection (***n*** = 31)	No pathogen (***n*** = 29)
**Sex (**male), n (%)	108 (70.6%)	28 (77.8%)	32 (56.1%)	24 (77.4%)	24 (82.7%)
**Age (**years), median (range)	62 (51–73)	65 (53–77)	61 (48–68)	62 (57–73)	62 (51–73)
**SAPS II**, median (range)	37 (27–47)	44.5 (34–53.5)	33 (27–44)	42 (25–55.5)	31 (19–41)
**BMI**, median (range)	27 (23–32)	25 (23–27)	31 (26.5–35)	27 (23–31)	28 (23–31)
**Seasonal influenza vaccination**, n (%)	47 (30.7%)	16 (44.4%)	12 (21%)	8 (25.8%)	11 (37.93%)
**Pneumococcal vaccination**, n (%)	27 (17.6%)	8 (22.2%)	5 (8.8%)	9 (29%)	5 (17.24%)
**COPD**, n (%)	37 (24.2%)	6 (16.7%)	11 (19.3%)	7 (22.6%)	13 (44.83%)
**Asthma**, n (%)	9 (5.9%)	1 (2.8%)	5 (8.8%)	2 (6.4%)	1 (3.4%)
**Chronic heart failure**, n (%)	22 (14.4%)	1 (2.8%)	10 (17.5%)	7 (22.6%)	4 (13.8%)
**Chronic renal failure**, n (%)	12 (7.8%)	2 (5.6%)	5 (8.8%)	2 (6.4%)	3 (10.3%)
**Diabetes**, n (%)	27 (17.6%)	3 (8.3%)	10 (17.5%)	8 (25.8%)	6 (20.7%)
**Tobacco use**, n (%)	57 (37.2%)	13 (36.1%)	21 (58.3%)	10 (32.3%)	13 (44.8%)
**Alcohol abuse**, n (%)	29 (18.9%)	9 (25%)	5 (8.8%)	10 (32.3%)	5 (17.2%)
**Immunocompromised patient**, n (%)	41 (26.8%)	11 (30.6%)	11 (19.3%)	12 (38.7%)	7 (24.1%)
**Patient treated with antibiotic(s)**, n (%)	152 (99.3%)	36 (100%)	57 (100%)	31 (100%)	28 (96.5%)
**Patient treated with antiviral drug(s)**, n (%)	21 (13.7%)	0 (0%)	18 (31.6%)	3 (9.7%)	0 (0%)
**Patient mechanically ventilated**, n (%)	113 (73.9%)	33 (91.7%)	39 (68.4%)	20 (64.5%)	21 (72.4%)
**Patient with tracheal intubation**, n (%)	89 (58.2%)	30 (83.3%)	28 (49.1%)	19 (61.3%)	12 (41.4%)
**Invasive mechanical ventilation** (day), median (range)	7 (4–14)	7 (5–9.7)	8 (4–15.7)	6.5 (4.2–10.7)	7 (5.2–11.7)
**Non-invasive mechanical ventilation** (day), median (range)	2 (1–4)	2 (2–4)	2 (1–4.5)	1 (1–1)	2.5 (1–3)
**ARDS**, n (%)	41 (26.8%)	15 (41.7%)	13 (22.8%)	8 (25.8%)	5 (17.2%)
**Patient treated with vasopressor**, n (%)	47 (30.7%)	17 (47.2%)	13 (22.8%)	12 (38.7%)	5 (17.2%)
**Vasopressor** (day), median (range)	3 (2–5)	3 (2–5)	2 (2–3)	3.5 (1–6)	3 (2–3)
**Creatinine (μM)**, median (range)	106 (81–161)	113 (86–194)	108 (77–174)	121 (90–160)	88 (73–110)
**Renal replacement therapy**, n (%)	14 (9.1%)	7 (19.4%)	5 (8.8%)	1 (3.2%)	1 (3.4%)
**ICU mortality**, n (%)	13 (8.5%)	5 (13.9%)	3 (5.3%)	5 (16.1%)	0 (0%)

*SAPSII* Simplified acute physiology score II, *BMI* Body mass index, *COPD* Chronic obstructive pulmonary disease, *ARDS* Acute respiratory distress syndrome, *ICU* Intensive care unit

### Clinician predictions

Experts had “high” confidence in their predicted etiology only 18.8% of the time. Confidence levels were typically “moderate” (38.9%) or “low” (42.6%), but never “very low”. All three experts agreed in 61.1% of the cases. Correct predictions were made 66.7% of the time. The clinician predictions had a sensitivity of 0.86, specificity of 0.54, PPV of 0.54 and NPV of 0.86 for the diagnosis of bacterial pneumonia (Table 3). The LR+ for diagnosing a viral pneumonia was 3.81, and the corresponding LR- was 0.53. The LR+ for diagnosing a bacterial pneumonia was 1.89, and the corresponding LR- was 0.26. Therefore, the discriminant abilities of experienced physicians to distinguish viral and bacterial etiologies for pneumonia were categorized as *very low* to *low* (according to defined cutoff values for the interpretation of likelihood ratios, see Table 1).

**Table 3:** Diagnostic prediction performances

	Data-driven approach predictions			Clinician predictions
	Algorithm built from clinical data	Algorithm built from biological and radiological data	Algorithm built from all data sources	
**Sensitivity**	0.75	0.54	0.57	0.86
**Specificity**	0.71	0.86	0.91	0.54
**PPV**	0.43	0.86	0.80	0.54
**NPV**	0.91	0.54	0.77	0.86
**Accuracy**	0.72	0.67	0.78	
**AUC**	0.72	0.81	0.84	
**LR + bacterial pneumonia**	2.62	3.82	6.29	1.89
**LR + viral pneumonia**	2.86	1.89	2.12	3.81
**LR - bacterial pneumonia**	0.35	0.53	0.47	0.26
**LR - viral pneumonia**	0.38	0.26	0.16	0.53

*PPV* Positive predictive value, *NPV* Negative predictive value, *AUC* Area under the curve, *LR* Likelihood ratio

#### Data-driven approach predictions

**Fig. 3:**
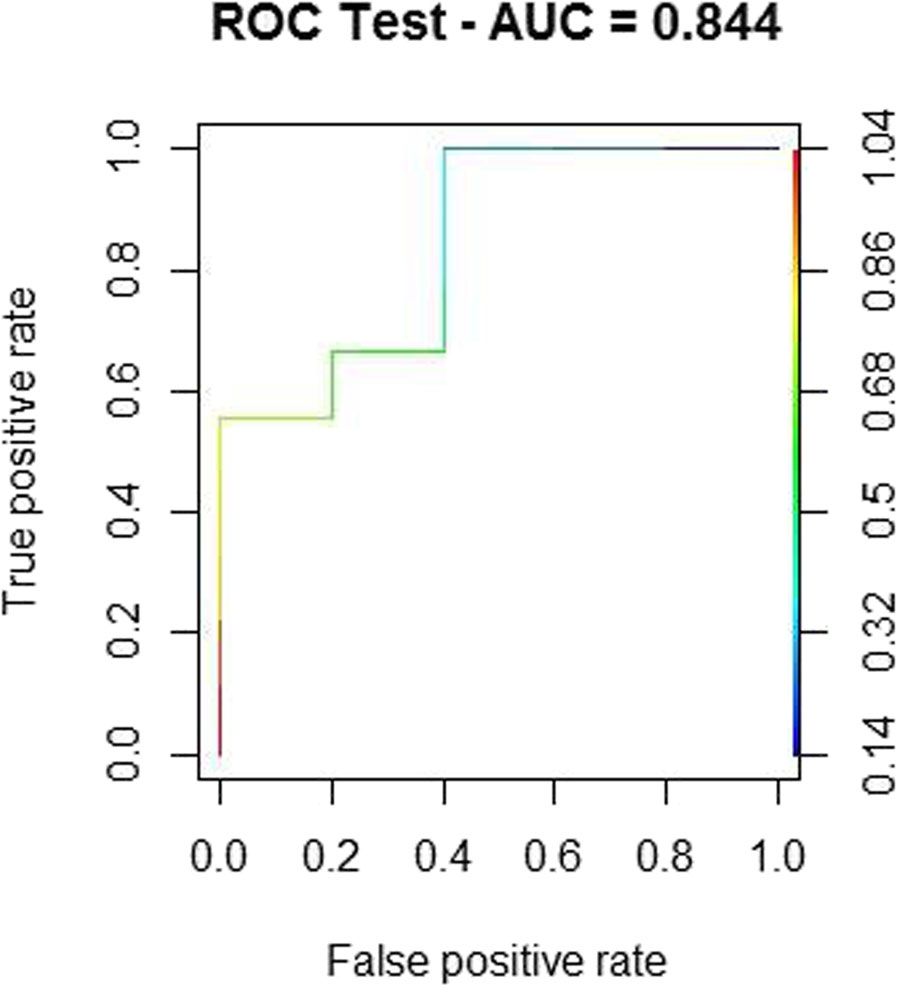
ROC curve of the data-driven algorithm predictions

Predictions by the data-driven algorithms generated from clinical data alone resulted in an ROC curve with a corresponding AUC of 0.72. Predictions by the data-driven algorithms generated from biological and radiological variables data alone resulted in an ROC curve with an AUC of 0.81. Finally, predictions generated from the dataset that included all data sources outperformed the other algorithms and resulted in an ROC curve with an AUC of 0.84 (Table 3, Fig. 3). This model based on the more inclusive dataset was considered the final model for comparison with the expert panel. The final algorithm made predictions with a sensitivity of 0.57, specificity of 0.91, PPV of 0.80 and NPV of 0.77 for the diagnosis of bacterial pneumonia. The LR+ for diagnosing a viral pneumonia was 2.12, and the corresponding LR- was 0.16. The LR+ for diagnosing a bacterial pneumonia was 6.29, and the corresponding LR- was 0.47. Consequently, the discriminant abilities of the data-driven algorithm to distinguish viral and bacterial etiologies for pneumonia were categorized as *low* to *moderate* (according to defined cutoff values for the interpretation of likelihood ratios, see Table 1).

## Discussion

Addressing antimicrobial resistance requires investment in several critical areas, the most pressing of which is the ability to make rapid diagnoses to promote appropriate anti-infective therapeutics and limit unnecessary antibiotic use. Here, we set up a pilot study and demonstrated that neither experts nor a mathematical algorithm could accurately predict the microbial etiology of severe CAP within the first 3 hours of hospitalization when there is an urgent need to define the appropriate anti-infective therapeutic strategy.

We encoded all information available in the first 3 hours after admission for a large cohort comparable with other published cohorts in terms of the distribution of causal microbial pathogens, patient characteristics and severity of disease [15–17]. We demonstrated that experienced clinicians synthesizing all this information failed to adequately answer the question: “*is it a viral or a bacterial pneumonia?*”, as the discriminant ability between the two diagnoses was considered *low*. We interpreted our results mainly based on the calculation of likelihood ratios, as recommended for reports of a diagnostic test for an infectious disease [12]. Likelihood ratios incorporate both sensitivity and specificity and, unlike predictive values, do not vary with prevalence, making them good statistical tools to facilitate translation of knowledge from research to clinical practice [12]. In parallel, we designed a data-driven approach. Different AI methods were available; we selected the Random Forest method because it is one of the most efficient strategies for providing a predictive algorithm in this context [18–21]. Importantly, the final algorithm was tasked with providing predictions for a novel population independent of the dataset used for the algorithm construction. The discriminant abilities of the AI approach restricted to the binary choice “viral” or “bacterial” were superior to those of experts but still considered *low* or *moderate* and were ultimately insufficient to provide an indisputable therapeutic decision. It is important to emphasize that we chose a high cutoff value for determining the discriminant ability of the AI approach (LR+ > 10, LR- < 0.1); this choice was made for two reasons. First, in this proof-of-concept study, we did not analyze co-infections and restricted the possible choices to a binary prediction. Because we reduced the complexity of the cases, we expected high predictive performances. Second, the goal of this study was not a prediction of outcomes (e.g., ICU length of stay, mortality), which are informative but do not determine patient management; it was to provide a clear and immediate medical decision: whether or not to prescribe antibiotics. The immediate clinical consequences in this situation demand a high predictive performance. Still, it is important to highlight that the machine learning method we developed achieved an AUC of 0.84, which is superior or at least equal to AUC values usually observed for predictive mathematical models developed for the ICU environment. For instance, the Systemic Inflammatory Response Syndrome (SIRS) criteria, the Simplified Acute Physiology Score II (SAPS II) and the Sequential Organ Failure Assessment (SOFA) have AUC values of 0.61, 0.70 and 0.73, respectively, for identifying sepsis [22]. An AI Sepsis Expert algorithm for early prediction of sepsis has been engineered and achieved AUC values ranging from 0.83–0.85 according to the time of the prediction. An AI method for predicting prolonged mechanical ventilation achieved an AUC of 0.82 [23].

How can it be that AI or machine-learning predictive algorithms that can already automatically drive cars or successfully understand human speech failed to predict the microbial cause of pneumonia accurately? First, having data of excellent quality is critical for the success of AI predictions. The ICU environment is data-rich, providing fertile soil for the development of accurate predictive models [24], but it is also a challenging environment with heterogeneous and complex data. In our study, the data that fueled the AI method were from a real-world data source. It is probably more difficult to create a consistent data format when merging data from interviews, patient examinations, biological and radiological information than when using datasets from the insurance or finance industries. Additionally, data arising from patient examinations and interviews are still strictly dependent on the physician’s skill and experience. Finally, although we hypothesized that the AI capabilities would exceed human skills and make accurate predictions when physicians cannot, we must also consider the null hypothesis: viral and bacterial pneumonias share the same characteristics and cannot be distinguished based on initial clinical, biological or radiological parameters. The dividing lines between the signs and symptoms of a viral versus a bacterial infection could be too blurry to permit the two diagnoses to be discerned without microbial analyses.

Our results emphasize the need to use a rapid turnaround time system for the accurate identification of respiratory pathogens from patient specimens. Utilizing rapid molecular respiratory panel assays may increase the likelihood of optimal treatment of acute respiratory infections [25–29]. However, antibiotic consumption was not reduced by the use of a molecular point-of-care strategy in adults presenting with acute respiratory illness in a large randomized controlled trial [28]. It seems that we are experiencing a switch in perspectives regarding microbial diagnoses of respiratory infections: physicians are used to dealing with an absence of information, but they will likely be overloaded with information in the near future [30]. The positive detection of respiratory viruses may or may not be useful for the immediate management of a patient [31]. Thus, the development of molecular point-of-care analysis techniques will not lessen the usefulness of our AI strategy. On the contrary, we believe that AI could be a great help in dealing with information overload, which could soon be a common problem. AI methods should not be viewed as ways to replace human expertise but rather as catalysts that accelerate human expertise–based analyses of data. AI methods can assist–rather than replace–in clinical decision-making by transforming complex data into more actionable information. Further studies are needed to assess if AI system integrated with point-of-care rapid molecular respiratory panel assays could be a useful addition for the clinician. Ultimately, randomized controlled trial should determine the effect of this strategy on the decision making regarding antibiotic use.

Our study should be interpreted in the context of several limitations. First, this was a proof-of-concept study, and we excluded cases of CAP with mixed etiology or without microbiological documentation. Consequently, the results were obtained from artificially dichotomized situations (viral *or* bacterial pneumonia, 93 patients in total) and cannot be directly extrapolated to real-life practice. Moreover, we did not include cases of acute pneumonia with non-infectious origins. Second, the experts were asked to make their predictions based on case reports exhaustively described in Excel files. They did not have the opportunity to interview or directly examine the patients themselves. Furthermore, the experts’ predictions were not performed in “real-life” situation. This could have affected the experts’ predictive performance. Third, we cannot rule out the possibility that some bacterial or viral pneumonia cases were misdiagnosed. We relied on state-of-the-art methods for microbial discovery, but it is possible that our current technology is sometimes suboptimal for detecting respiratory microbial pathogens.

## Conclusion

Neither a panel of experts nor a data-driven approach could accurately distinguish viral from bacterial pneumonia within the first hours of patient admission in ICU for CAP. The heterogeneous and complex data generated in the ICU environment are likely difficult to use to generate an AI algorithm with a high predictive quality. The results of our pilot study at least highlight that we should not treat machine learning and data science as crystal balls for making predictions and automating decision-making; we should rather use these techniques to more critically examine all available information and enhance existing human expertise.

## Supplementary information

**Additional file 1: Supplementary Table S1.** Prospective data collection of elements available in the first 3-hour period after admission. Clinicians and a mathematical algorithm were tasked with predicting the microbial etiology of pneumonia cases based on this information. Qualitative data were processed as binary information (i.e. influenza immunization: present “1”, absent “0”). Raw data were provided for quantitative values (no cut-offs defined).

## Data Availability

The datasets used and/or analysed during the current study are available from the corresponding author on reasonable request.

## Abbreviations

AHRQ: Agency for Healthcare Research and Quality
AI: Artificial Intelligence
AUC: Area Under the Curve
BAL: Broncho-Alveolar Lavage
CAP: Community-Acquired Pneumonia
ICU: Intensive Care Unit
IQR: InterQuartile Range
LR: Likelihood Ratios
NPV: Negative Predictive Value
PPV: Positive Predictive Value
ROC: Receiver Operating Characteristic
SIRS: Systemic Inflammatory Response Syndrome
SOFA: Sequential Organ Failure Assessment
WHO: World Health Organization
