# Supplementary material for: Predicting the microbial cause of community-acquired pneumonia: can physicians or a data-driven method differentiate viral from bacterial pneumonia at patient presentation?
Source: BMC Pulm Med. 2020 Mar 6;20:62. doi: 10.1186/s12890-020-1089-y (PMC7060632; doi:10.1186/s12890-020-1089-y)
Supplement: Supplementary file 1 — Additional file 1: Supplementary Table S1. Prospective data collection of elements available in the first 3-hour period after admission. Clinicians and a mathematical algorithm were tasked with predicting the microbial etiology of pneumonia cases based on this information. Qualitative data were processed as binary information (i.e. influenza immunization: present “1”, absent “0”). Raw data were provided for quantitative values (no cut-offs defined). [file 12890_2020_1089_MOESM1_ESM.docx]

**Supplementary Table 1.** **Prospective data collection of elements available in the first 3-hour period after admission**. Clinicians and a mathematical algorithm were tasked with predicting the microbial etiology of pneumonia cases based on this information. Qualitative data were processed as binary information (i.e. influenza immunization: present “1”, absent “0”). Raw data were provided for quantitative values (no cut-offs defined).

| **Patients ‘characteristics and comorbidities** | **Patient semi-structured interview questions** | **Clinical examination** | **Biological findings** | **Chest radiography** |
| --- | --- | --- | --- | --- |
| Gender  Age  Body mass index  Influenza immunization (within 1 year)  Pneumococcal immunization (within 5 years)  Chronic obstructive pulmonary disease  Asthma  Ischemic heart disease  Heart failure  Chronic kidney disease  Diabetes mellitus  Smoking status  Alcohol dependence  Immunosuppression | Time between the onset of symptoms and hospitalization  Presence before admission of: Fever,  sweats,  chills,  myalgia or arthralgia,  cough,  sore throat or nasal congestion,  chest pain,  hemoptysis,  difficulty or shortness of breath,  nausea, vomiting or abdominal pain  Exposure to ill persons  Medication before admission with:  corticosteroid,  non-steroid anti-inflammatory drugs,  antibiotic therapies | Glasgow coma scale  Body temperature  Heart rate  Systolic, diastolic and mean arterial blood pressures  Marbling  Cyanosis  Respiratory rate*  Oxygen saturation (SpO_2_ < 85% or > 8l/mn)  Asymmetrical respiratory sounds or abnormalities  Inspiratory crackles  Wheezes  Rhonchi | White-cell count  Neutrophil count  Lymphocyte count  Platelet count  Lactate dehydrogenase*  Troponin*  Creatinine  Creatine kinase*  Lactate  Bilirubin  Aspartate aminotransferase*  Alanine aminotransferase  Procalcitonin  C - reactive protein | Unilateral or bilateral abnormalities  Interstitial opacities  Consolidations |

*indicate element with missing values between 5 to 10%. No element had more than 10% of missing values
